# Foci of cyclin A2 interact with actin and RhoA in mitosis

**DOI:** 10.1038/srep27215

**Published:** 2016-06-09

**Authors:** Abdelhalim Loukil, Fanny Izard, Mariya Georgieva, Shaereh Mashayekhan, Jean-Marie Blanchard, Andrea Parmeggiani, Marion Peter

**Affiliations:** 1 Institut de Génétique Moléculaire de Montpellier, CNRS, Université de Montpellier, 1919 route de Mende, 34293 Montpellier, France; 2 Dynamique des Interactions Membranaires Normales et Pathologiques, CNRS, Université de Montpellier, Pl. E. Bataillon, 34095 Montpellier Cedex 5, France; 3 Laboratoire Charles Coulomb, CNRS, Université de Montpellier, Pl. E. Bataillon, 34095 Montpellier Cedex 5, France

## Abstract

Cyclin A2 is a key player in the regulation of the cell cycle. Its degradation in mid-mitosis depends primarily on the ubiquitin-proteasome system (UPS), while autophagy also contributes. However, a fraction of cyclin A2 persists beyond metaphase. In this work, we focus on cyclin A2-rich foci detected in mitosis by high resolution imaging and analyse their movements. We demonstrate that cyclin A2 interacts with actin and RhoA during mitosis, and that cyclin A2 depletion induces a dramatic decrease in active RhoA in mitosis. Our data suggest cyclin A2 participation in RhoA activation in late mitosis.

Cyclin A2 is an essential regulator of the cell cycle^1^, interacting with Cyclin-dependent kinases 1 and 2 (Cdk1, Cdk2), and thereby involved in mitosis and interphase^2^. Cyclin A2 degradation occurs in mitosis and is required for chromosome alignment and anaphase progression^3^,^4^. While most cyclin A2 is degraded before metaphase by the ubiquitin-proteasome system (UPS)^3^,^4^,^5^, we have recently shown that a small but significant fraction of cyclin A2 persists beyond metaphase and that autophagy also mediates cyclin A2 degradation^6^.

Moreover, our laboratory has shown that cyclin A2 is also involved in cell invasion *via* RhoA signalling^7^,^8^,^9^. The Rho family members RhoA, Rac1 and Cdc42 are small GTPases that regulate cell morphology, motility and cytokinesis, mainly through reorganisation of actin filaments. Cyclin A2 promotes RhoA activation by potentiating the exchange activity of a RhoA-specific guanine nucleotide exchange factor (GEF). Consistent with this, cyclin A2 depletion impaired RhoA activation and resulted in increased cell motility and invasiveness^7^. The precise localisation of RhoA at the cortical regions of the cleavage furrow, seems to be essential to coordinate cytokinesis. RhoA activity is increased at the end of mitosis and its depletion prevents cleavage furrow formation^10^,^11^,^12^. The GEFs Ect2 and GEF-H1 modulate, respectively, RhoA localisation and activation at the cleavage site^13^.

Actin is dramatically reorganised in mitosis, inducing cell rounding with increased cortical rigidity. Actin filaments form an amorphous cluster that appears in prometaphase, revolves along the cell cortex and fuses into the contractile ring. Cdk1 activity and the Arp2/3 complex are essential for the formation of this cluster and its movements^14^. Interestingly, actin depolymerisation by latrunculin blocks centrosome splitting in interphase and causes mitotic delay and cytokinesis failure^15^. Moreover, pharmacological stabilisation and delocalisation of filamentous actin (F-actin) induces cytokinesis defects and multinuclear cells^16^. Knock-down studies of actin-related proteins have shown multiple defects affecting mitosis, as well as cell cycle progression^17^.

When studying cyclin A2 in mitosis in human cells, we identified a novel cyclin A2-containing compartment that forms dynamic foci^6^. Here, we analyse the movement of cyclin A2 foci during mitosis using high resolution microscopic imaging and data analysis. This revealed that cyclin A2 interacts with actin and RhoA in mitosis.

## Results and Discussion

### Cyclin A2-rich foci persist until telophase and localise to the cleavage furrow

**Figure 1:**
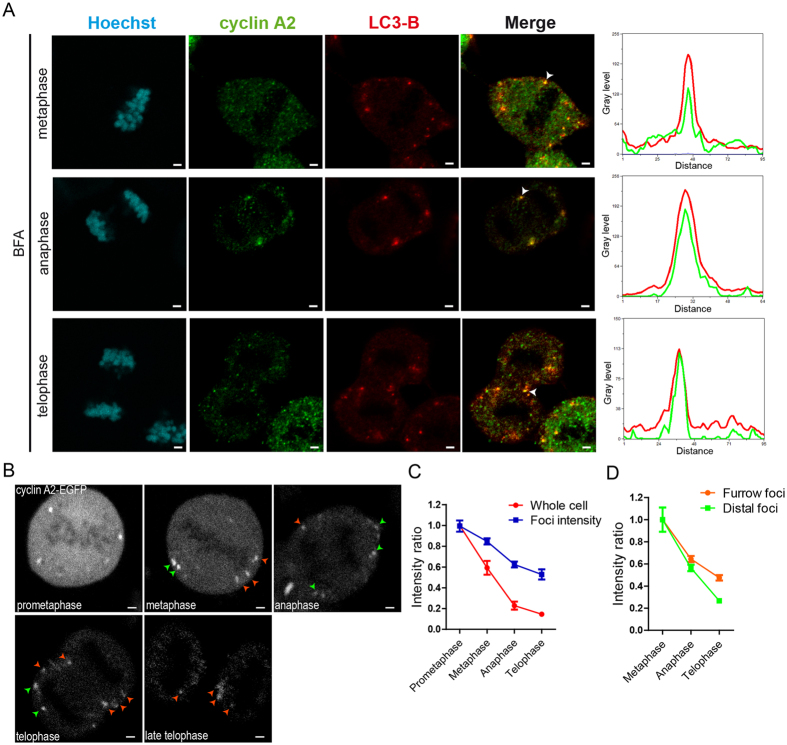
Cyclin A2-rich foci persist until telophase and localise to the cleavage furrow. (**A**) Observation of endogenous cyclin A2 foci. MCF-7 cells were treated with bafilomycin A1 (BFA), fixed and immunostained for cyclin A2 and LC3-B. Confocal images. Spectral colocalisation plots of the indicated foci (arrowheads) are shown to the right. Representative images of >50 cells from three independent experiments. Scale bars: 2 μm. (**B**) Imaging of cyclin A2-EGFP foci. A MCF-7 cell synchronised by single thymidine block, microinjected with pEGFP-N1-cyclin A2 and observed in mitosis 14 h after release. Images obtained by two-photon excitation with a FLIM detector. Green and orange arrows point to distal and furrow foci, respectively. Representative images of >100 cells from three independent experiments. Scale bars: 2 μm. (**C,D**) Quantification of EGFP intensity in images of 4 cells (103 foci in total) followed during mitosis (as in (**B**)). The data shown in the graph represent the mean intensity relative to *t*_*0*_ (prometaphase in (**C**), metaphase in (**D**)). Data representative of three independent experiments.

Cyclin A2-rich foci were detected two ways in different human cell lines, by immunostaining the endogenous protein (Fig. 1A and ref. 6) and following expression of cyclin A2 fused to EGFP (enhanced green fluorescent protein) after microinjection or stable transfection with an inducible vector (Fig. 1B and ref. 6). These foci were observed between prometaphase and telophase (Fig. 1A,B and ref. 6). Their detection was very difficult using confocal microscopy and easier by accumulating photons through time on a FLIM (fluorescence lifetime imaging microscopy) detector, even though the GaAsP (gallium arsenide phosphide) detector used for confocal images and the FLIM detector were of comparable sensitivity. Confocal images were single snapshots to limit photobleaching. FLIM acquisitions were longer, using two-photon excitation to keep photobleaching and photodamage to a minimum, and FLIM images were calculated from the integrated number of photon counts for each pixel. We thus used confocal microscopy for immunodetection of endogenous cyclin A2 and colocalisation experiments (Fig. 1A), and imaging with two-photon excitation and a FLIM detector for time-lapse experiments with cyclin A2-EGFP expressing cells (Fig. 1B).

Both endogenous and exogenous (EGFP-tagged) cyclin A2 foci displayed similar localisation during mitosis (Fig. 1A,B). In prometaphase, cyclin A2-rich compartments were detected mainly at the periphery of the cell, close to the membrane. In metaphase, some cyclin A2 foci were detected in the equatorial plane, lateral to the aligned chromosomes. In anaphase and telophase, some cyclin A2 foci were observed on both sides of the cleavage furrow, at equivalent levels in the daughter cells, while others appeared distal.

To further characterise cyclin A2-containing foci during mitosis, we immunostained the endogenous protein in MCF-7 cells (Fig. 1A). As autophagy contributes to cyclin A2 degradation, cells were treated with bafilomycin A1 (BFA) to inhibit this pathway and then immunostained for light-chain 3-B protein (LC3-B), a marker of autophagosomes. Several cyclin A2 foci were colocalised with LC3-B in metaphase, anaphase and telophase. Importantly, most foci co-stained for cyclin A2 and LC3-B localised to the equatorial plane in metaphase and anaphase, and to the cleavage furrow region in telophase (Fig. 1A).

To investigate the overall behaviour of cyclin A2-EGFP in the whole cell versus the enriched compartments, the EGFP intensity was measured during mitosis (Fig. 1C). The EGFP intensity decreased more rapidly in the whole cell than in the foci, indicating a differential degradation of cyclin A2-EGFP during mitosis. In the whole cell, the fraction remaining of cyclin A2-EGFP in anaphase and telophase was about 22% and 16%, respectively, while it was 62% and 50%, respectively, in cyclin A2 foci (Fig. 1C). Furthermore, we measured EGFP intensity in foci with different localisation in late mitosis, either close to the cleavage furrow (furrow foci) or not (distal foci). In anaphase and telophase, the EGFP intensity decreased less in furrow foci than in distal foci (Fig. 1D).

Only the foci localised to the cleavage furrow were still detectable in late telophase. This localisation was also observed with cyclin A2-EGFP foci in U2OS cells stably transfected with an inducible expression vector (Supplementary Figure S1). The persistence of a fraction of cyclin A2 in late mitosis observed here by imaging is in agreement with western blot experiments performed with extracts from synchronised cells^6^.

### Cyclin A2-rich foci exhibit distinct dynamic populations

**Figure 2:**
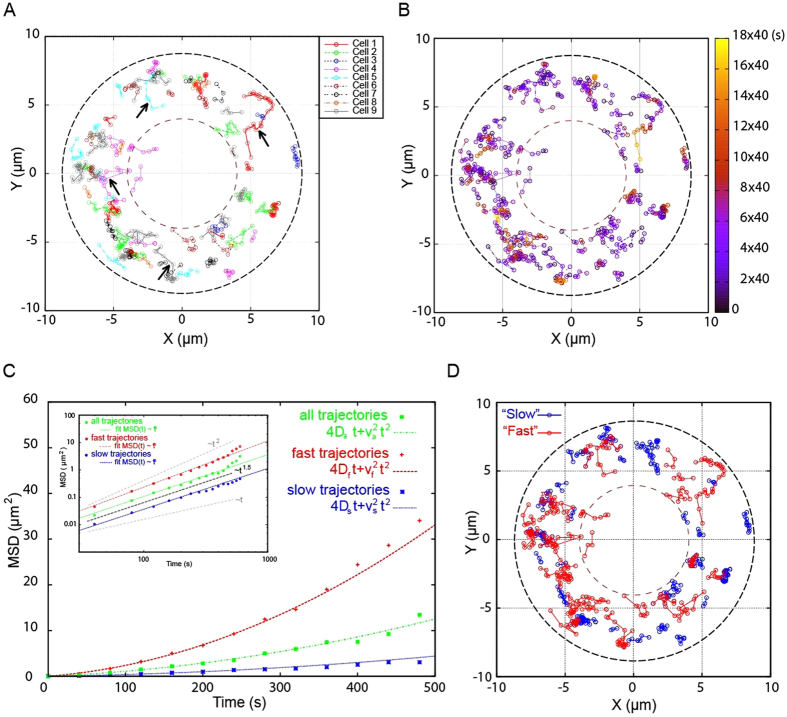
Foci trajectory analysis. (**A**) Superimposition of 77 cyclin A2-EGFP foci trajectories observed in nine cells, between prometaphase and metaphase, by time-lapse with a FLIM detector. The dashed lines emphasise the region of foci movement in the focal plane. This region is confined externally by the cell boundary and cortex, and internally by the region occupied by the chromosomes and mitotic machinery. Trajectories have different lengths probably because of foci exit from the focal plane. Arrows point to long trajectories suggesting a directed motion of the foci. (**B**) Time evolution of foci trajectories depicted in (**A**). Trajectories display stochastic motions and orientations, but many of them display also directed motion. (**C**) Mean square displacement (MSD) time analysis. Inset: log-log representation of MSD in time. Linear behaviour in log-log scale corresponds to a power law behaviour proportional to t^α^ in linear coordinates. These data suggest that foci motion can be super-diffusive, with exponent 1 ≤ α~1.5 ≤ 2. Interestingly this exponent is close to observations of organelle super-diffusive motion in amoeba^19^. MSD analysis was performed also *via* a fit with the typical expression of MSD time dependence *via* a biased Brownian motion, with a typical diffusion coefficient D and directed displacement defined by an average velocity v, MSD ~ 4Dt + v^2^t^2^. D and v values extracted from data analysis are compatible with *in vivo* dense actin solutions in presence of active cytoskeletal processes (such as motor protein activity and actin assembly and disassembly)^20^,^21^. Moreover, foci seem to be distributed in two populations, with diffusion constants and average velocities that can differ by a factor 5 and 3 respectively. (**D**) Foci trajectories classified as “fast” or “slow” *via* the MSD analysis.

To investigate cyclin A2-rich foci movement, live MCF-7 cells expressing cyclin A2-EGFP were imaged by time-lapse FLIM during mitosis (Fig. 2A,B and Supplementary movie 1). We superimposed images of different cells to track 77 foci between prometaphase and metaphase. After metaphase, the number and fluorescence intensity of foci decreased, rendering their detection more difficult. The superimposition was possible due to the similar round shape and size (about 18 μm) of mitotic cells. Moreover, the chromosome alignment axis provided a useful reference to extract foci movements with respect to the cell frame. Figure 2A shows the superimposition of all foci movements taken in the focal plane. Interestingly, foci are localised in the region outside the volume occupied by chromosomes during mitosis, but they are also peripheral, close to the plasma membrane. Different kinds of trajectories are observed. Some of them show Brownian diffusion-like motion, while others clearly display directed motion, some several micrometers long (arrows in Fig. 2A). These behaviours are summarised in Fig. 2B, where focal progression in time is depicted by a time-lapse colour code.

A more detailed analysis of foci trajectories allowed us to quantify focal dynamics during mitotic progression. The analysis of the mean squared displacement time dependence (MSD)^18^ suggests that foci move super-diffusively^19^,^20^ or with biased Brownian motion (Fig. 2C and Table 1). In particular, we found that foci can be split in two populations, one (29 out of 77 foci) moving with a typical diffusion constant and directed average speed larger than the second population (Table 1 and Fig. 2D). Diffusion constants for both foci populations are, in general, very small, suggesting that motion takes place in a highly viscous environment (between 10^2^–10^3^ fold water viscosity) (see Supplementary material). Remarkably, even in the presence of high effective viscosity, foci move with directed motion at a speed compatible with actin cytoskeleton dynamics^19^,^20^,^21^. Furthermore, the analysis of the radial and angular speed distributions, as well as the associated fluxes of foci, confirms the presence of faster (probably driven) movements coupled with slower or purely diffusive Brownian motion (see Supplementary material.

Altogether, our observations suggest that the recorded foci movements can be the result of cytoplasm dynamics, with very high viscosity reflecting a direct or indirect interaction with the cell cytoskeleton. The following experiments are therefore focused on the biochemical characterisation, *in vitro* and in cells, of the potential interaction between cyclin A2 and the actin cytoskeleton.

### Cyclin A2 interacts with actin in mitosis

**Figure 3:**
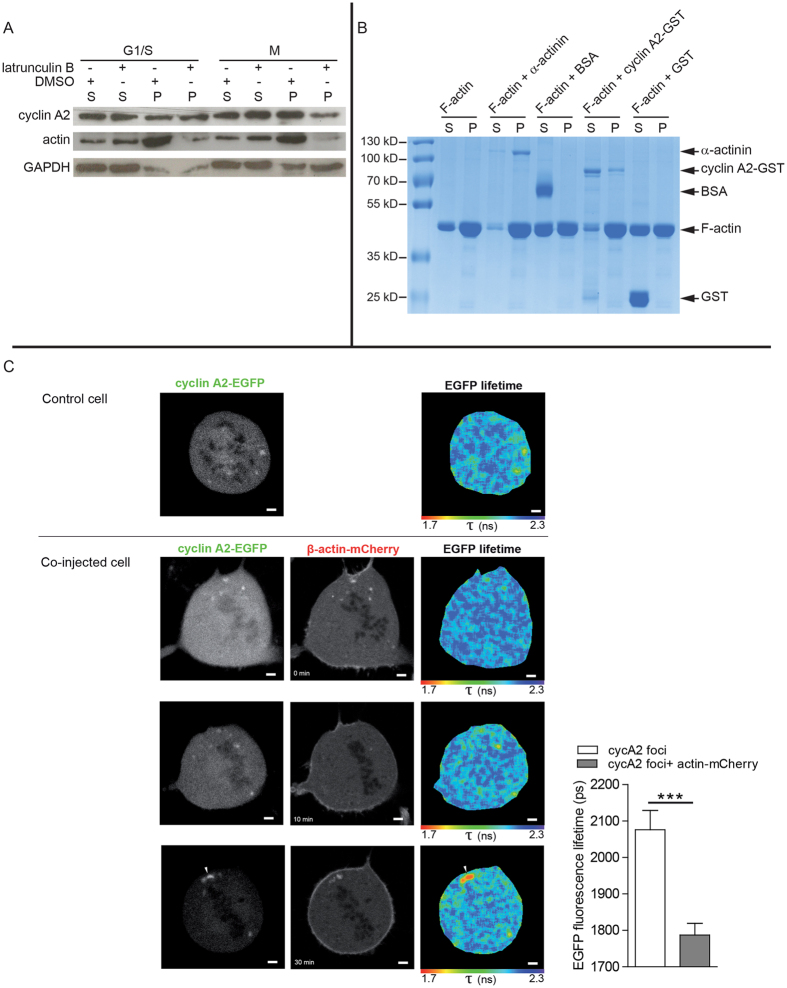
Cyclin A2 interacts with actin in mitosis. (**A**) U2OS cells were synchronised in G1/S or M, treated with latrunculin B or DMSO and fractionated. Immunoblots showing cyclin A2 and actin levels in the soluble (SN: supernatant) and insoluble (P: pellet) fractions. GAPDH is used as a loading control. (**B**) Spin-down assay with F-actin using purified cyclin A2-GST. α-actinin is used as positive control, BSA and GST as negative controls. Coomassie blue staining following SDS-PAGE. Data shown in (**A**,**B**) are representative of three independent experiments. (**C**) Left panels, live MCF-7 cells microinjected with pEGFP-N1-cyclin A2, with or without pmCherry-C1-β-actin. The co-injected cell is followed through time. Two-photon EGFP images and corresponding EGFP lifetime maps. mCherry confocal images. Representative images of 10 cells (100% showing the same transient interaction) analysed in two independent experiments. Scale bars: 2 μm. Right panel, data represent the mean ± s.e.m. of EGFP lifetimes in cyclin A2-EGFP foci (n = 40, P < 0.0005).

To test whether cyclin A2 interacts with actin, we first treated U2OS cells synchronised in G1/S or in mitosis (M) with latrunculin B, to induce depolymerisation of filamentous actin (F-actin). We then analysed cyclin A2 levels in the soluble and insoluble fractions. Following latrunculin B treatment, actin levels were decreased in the insoluble fraction of both samples, as expected (Fig. 3A). Interestingly, cyclin A2 was decreased in the insoluble fraction of cells synchronised in M, but not in G1/S, suggesting its interaction with F-actin in mitosis (Fig. 3A and Supplementary Figure S3).

We next performed an *in vitro* binding assay between F-actin and purified cyclin A2-GST (glutathione S-transferase), using an Actin Binding Protein Biochem Kit (Cytoskeleton). After ultracentrifugation, 37% of the purified cyclin A2-GST was detected in the pellet with F-actin (Fig. 3B), versus undetectable binding by GST or BSA (bovine serum albumin).

Furthermore, we investigated cyclin A2 interaction with actin in live cells by using FRET (Förster or fluorescence resonance energy transfer) measured by FLIM. Synchronised MCF-7 cells were microinjected with vectors encoding cyclin A2-EGFP and β-actin-mCherry. As expected, β-actin-mCherry displayed a cortical localisation in mitosis. In metaphase, we observed FRET between cyclin A2-EGFP and β-actin-mCherry in foci located at the cell periphery, in the equatorial plane (Fig. 3C, bottom row).

Thus our data show that a fraction of cyclin A2 interacts with F-actin in mitosis, a novel finding to our knowledge. In the literature, other interactions between cyclin A and actin-related proteins have been identified by quantitative proteomics in G2 phase^22^. However, this study did not include mitotic cyclin A complexes, which could explain why actin was not detected as a partner.

### Cyclin A2 interacts with RhoA in mitosis

Rho GTPases elicit distinct effects on the actomyosin cytoskeleton to accurately promote cytokinesis^23^. Among them, RhoA is the central positive regulator of cytokinesis. At the division plane, RhoA stimulates both filamentous actin assembly and myosin-II motor activity to promote the assembly and constriction of a contractile ring^24^. Moreover, we previously showed that cyclin A2 directly interacts with RhoA *in vitro* and regulates its activation state by potentiating RhoA GEF exchange activity^7^.

**Figure 4:**
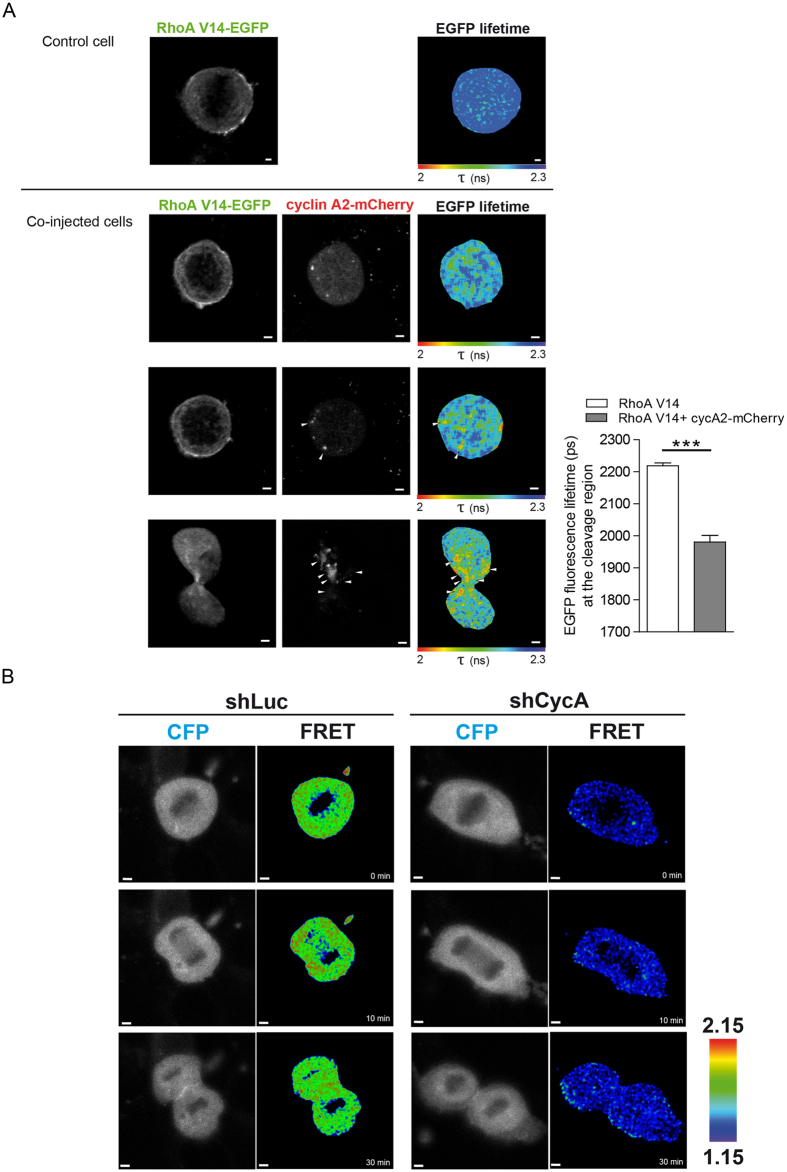
Cyclin A2 interacts with RhoA in mitosis. (**A**) Left panels, live MCF-7 cells microinjected with pEGFP-C-RhoA-V14, with or without pmCherry-N1-cyclin A2. The co-injected cell is followed through time. Two-photon EGFP images and corresponding EGFP lifetime maps. mCherry confocal images. Representative images of 30 cells (93% showing the same interaction localisation) from four independent experiments. Scale bars: 2 μm. Right panel, data represent the mean ± s.e.m. of EGFP lifetimes (P < 0.0005). Measurements were performed in the cleavage region either randomly (RhoA-V14) or at sites corresponding to cyclin A2-mCherry foci (RhoA-V14 + cycA2-mCherry). (**B**) NMuMG cells were infected with retrovirus carrying shRNA against luciferase (shLuc) or cyclin A2 (shCycA_1), transfected with pTRIEx-RhoA FLARE.sc Biosensor WT and imaged through mitosis. Wide field CFP images and FRET ratio images. Representative images of 60 cells (100% showing the same phenotype) obtained from three independent experiments. Scale bars: 2 μm.

To characterise the role of cyclin A2 foci in late mitosis, we investigated cyclin A2 interaction with RhoA in live mitotic cells by using FRET/FLIM. Synchronised MCF-7 cells were microinjected with vectors encoding the wild-type form (WT) or a constitutively active mutant form of RhoA, RhoA-V14-EGFP, and cyclin A2-mCherry. We observed FRET between RhoA-V14-EGFP and cyclin A2-mCherry in foci located in the area of the cleavage furrow in telophase (Fig. 4A, bottom row). However, we did not detect FRET between RhoA-WT-EGFP and cyclin A2-mCherry in mitosis (data not shown).

To study the functional link between cyclin A2 and RhoA in mitosis, we chose to image active RhoA with a fluorescent biosensor^25^ in cells expressing, or not, a short hairpin RNA (shRNA) targeting cyclin A2. In cells infected with virus encoding the control shRNA, active RhoA was found in mitotic cells, notably in foci located mostly at the cell periphery in anaphase, and close to the cleavage furrow in telophase (Fig. 4B). However, cyclin A2 depletion induced a dramatic decrease in active RhoA in mitosis (Fig. 4B and Supplementary Figure S4), suggesting that cyclin A2 foci participate in RhoA activation in late mitosis. This was confirmed with another shRNA targeting cyclin A2 (Supplementary Figure S4). Our results are consistent with the increase in mitotic duration in cyclin A2 depleted cells observed by our laboratory^6^,^7^ and by others^26^.

We thus propose that cyclin A2 foci, interacting with the actin cytoskeleton, would move towards the cleavage furrow, where cyclin A2 could participate in RhoA activation in telophase. Since RhoA is known to regulate actin, this could generate a positive feedback loop.

## Conclusions

Our work stresses the existence of cyclin A2 foci. In fact, many other intracellular bodies are being characterised nowadays. By colocalising molecules at high concentrations within small cellular micro-domains, they may provide functional advantages to the cells, such as catalytic efficiency or improved regulation^27^,^28^.

Autophagy has already been shown to promote the degradation of active RhoA^29^,^30^ and to be required for midbody ring disposal^31^. We can hypothesise that cyclin A2 would be degraded by autophagy when localised with the GTPase to the cortical region, whereas degradation of its soluble fraction would occur mainly through proteasomal activity.

While strengthening our previous observation of cyclin A2 as a potential modulator of RhoA activity, our results add another component to the complex regulatory network that involves cell cycle regulators and cytoskeletal structures that participate in the control of cell division. It is thus not surprising to find them involved in early and late steps of mitosis, when major cytoskeletal rearrangements occur. Our study identifies cyclin A2 and RhoA as key players in the control of both cell cycle and cell division, whose coordination – like the coordination between cell cycle and cell growth – remains to be investigated^32^,^33^.

## Methods

### Constructs

pEGFP-N1-cyclin A2, pLKO.1-Luciferase-shRNA and pSIREN-RetroQ-cyclin A2-shRNA vectors have been previously described^6^. Cyclin A2 targeting sequences are: shCycA_1:5′-GTAGCAGAGTTTGTGTATA-3′; shCycA_2: 5′-GAAATGGAGGTTAAATGA-3′. Human cyclin A2 cDNA was subcloned into pGEX-4T-1 (GE Healthcare) and pmCherry-N1^34^. Human β-actin cDNA was subcloned into pmCherry-C1^34^. pEGFP-C-RhoA-V14 was a kind gift of Cécile Gauthier-Rouvière (Centre de Recherche de Biochimie Macromoléculaire, Montpellier, France). pTRIEx-RhoA FLARE.sc Biosensor WT^24^ was obtained from Addgene.

### Cell culture, synchronisation, microinjection, infection, transfection and treatments

MCF-7 (human breast adenocarcinoma) and U2OS (human osteosarcoma) cells were grown in DMEM (Gibco) with 10% FBS (PAA). U2OS Tet-Off stable inducible cell line, expressing cyclin A2-EGFP^6^, was cultured with the same media supplemented with 250 μg/ml G418 (Sigma-Aldrich G9516), 200 μg/ml hygromycin (Calbiochem 400051) and 2 μg/ml tetracyclin (Sigma-Aldrich T7660). NMuMG (normal murine mammary gland) cells were grown in DMEM (Gibco) with 10% FBS (PAA) and 10 μg/ml insulin (Sigma).

Cell synchronisation in G1/S was performed by single thymidine block as described^6^. Cells were used at this stage, or washed and observed 14 h after release, in mitosis. Synchronisation in G2/M was performed by incubation with RO-3306 (9 μM for 20 h; 217699, Calbiochem). Cells were washed and observed 20 min after release, in mitosis.

DNA microinjection was performed into G1/S synchronised cells as described^6^.

NMuMG cells were infected with shRNA targeting Luciferase or cyclin A2. Cells were then selected using puromycin (InvivoGen) for 72 h. Lipofectamine 2000 was used to transfect the selected cells with pTRIEx-RhoA FLARE.sc Biosensor WT. 24 h later, mitotic cells were imaged.

Cells were treated with bafilomycin A1 (0.5 μM 3 h 30 min; B1793, Sigma-Aldrich) to inhibit autophagy flux. Cells were incubated with latrunculin B (5 μM for 45 min; 428020, Calbiochem) to depolymerise filamentous actin.

### Immunofluorescence

Cells were synchronised in G1/S by single thymidine block and observed 14 h after release, in mitosis. Cells were fixed and permeabilised with methanol/acetic acid. The following antibodies were used: cyclin A2 (clone 6E6, Novocastra Leica), LC3-B (L7543, Sigma-Aldrich), FITC-conjugated goat anti-mouse-IgG (731853, Cell Lab) and Alexa-Fluor-555-conjugated goat anti-rabbit-IgG (A21429, Invitrogen). Hoechst 33342 (Invitrogen) was used to stain DNA.

Samples were examined with a Zeiss LSM 780 microscope equipped with a Zeiss 63X/1.4 oil Plan-Apochromat objective. Images were captured using a GaAsP spectral detector interfaced with ZEN 2011 software. Images were processed using algorithms of MetaMorph 7.1 software. Image background was treated with “Background and Shading correction”. Merged images were obtained using “Color combine” and spectral colocalisation was performed using “Linescan”.

### FLIM-FRET

Time-domain FLIM was performed with a multiphoton microscopy system, based on a Zeiss LSM 510 Meta NLO equipped with a Ti: Sapphire Chameleon-XR pulsed laser (Coherent). Time-resolved detection was afforded by the addition at a non-descanned output of a fast hybrid photomultiplier (HPM-100-40) and SPC-830 time-correlated single-photon counting (TCSPC) electronics (Becker & Hickl). For EGFP excitation, laser power at 900 nm was adjusted to give average photon counting rates of the order 10^4^–10^5^ photons s^−1^ (0.0001–0.001 photons/excitation event) and with peak rates approaching 10^6^ photons s^−1^, below the maximum counting rate afforded by the TCSPC electronics to avoid pulse pile-up. FLIM acquisitions were performed on living mitotic cells (2 min acquisitions for single micrographs; 20 s acquisitions for the movie). Images were taken with a Zeiss 63X/1.4 oil Plan-Apochromat objective.

For the movie and foci trajectory study, images were aligned with ImageJ 1.44 software, using “StackReg” plugin (“Rigid body” option).

Analysis of the fluorescent transients was performed with SPCImage (Becker & Hickl) or TRI2 (Paul Barber, University of Oxford, UK).

### Numerical analysis of foci movement

Numerical data analysis of foci movements was performed using Excel (products.office.com). For the statistical analysis of the mean square displacement and the radial and angular speed and flux component distributions, we used Python programs (www.python.org) and fit routines from Gnuplot (www.gnuplot.info).

For the MSD analysis, we fitted the data with the typical expression of the MSD of biased Brownian motion in two dimensions. This allowed extracting typical values of diffusion coefficients and average speeds. Errors relative to the average speeds and diffusion coefficients were computed as asymptotic standard errors.

For the radial and angular speed distribution analysis, we fitted the speed histograms with two Gaussian functions in order to extract typical values of the speeds. Errors relative to the typical radial and angular speeds were computed as asymptotic standard errors.

### Immunoblotting

The following antibodies were used for immunoblotting: cyclin A (C4710, Sigma-Aldrich), actin (A5441, Sigma-Aldrich), GAPDH (G9545, Sigma-Aldrich). Secondary HRP-conjugated antibodies were obtained from Thermo Fisher Scientific.

### Actin spin-down assay

Cyclin A2-GST was produced in *Escherichia coli* and purified by standard procedures. Cyclin A2-GST binding to F-actin was assayed with the Actin Binding Protein Biochem Kit (BK013, Cytoskeleton) according to manufacturer instructions. Following SDS-PAGE and Coomassie staining, the gel was imaged with a ChemiDoc MP system (Biorad) and signals were quantified with Image Lab™ software.

### Intensity-based FRET

Cells were imaged with a Zeiss LSM 780 microscope equipped with a Zeiss 63X/1.4 oil Plan-Apochromat objective, with open pinhole. All acquisitions were performed in the same conditions and images displayed similar fluorescence intensities. FRET was analysed with ImageJ. The macro allowed background correction and intensity ratio calculation between the CFP (donor) and YFP (acceptor) images, pixel-by-pixel, generating a ratiometric FRET image.

### Statistical analysis

Data are reported as arithmetic means ± SEM. Statistical analyses were performed using nonparametric Mann-Whitney test with Prism software (GraphPad). Statistical significance was defined as P ≤ 0.05.

## Additional Information

**How to cite this article**: Loukil, A. *et al*. Foci of cyclin A2 interact with actin and RhoA in mitosis. *Sci. Rep.* **6**, 27215; doi: 10.1038/srep27215 (2016).

## Supplementary Material

Supplementary Information

Supplementary Movie 1

## Figures and Tables

**Table 1:** Mean square displacement analysis and estimate of typical diffusion coefficients and speeds.

Overall Population (77 foci)	Fast Population (29 foci)	Slow Population (48 foci)
D_a_ = (1.8 ± 0.4) · 10^−6^ μm^2^/s	D_f_ = (3.3 ± 0.6) · 10^−6^ μm^2^/s	D_s_ = (0.6 ± 0.1) · 10^−6^ μm^2^/s
v_a_ = (5.9 ± 0.7) · 10^−3^ μm/s	v_f_ = (10.3 ± 0.7) · 10^−3^ μm/s	v_s_ = (3.57 ± 0.26) · 10^−3^ μm/s
