# Supplementary material for: Foci of cyclin A2 interact with actin and RhoA in mitosis
Source: Sci Rep. 2016 Jun 9;6:27215. doi: 10.1038/srep27215 (PMC4899731; doi:10.1038/srep27215)
Supplement: Supplementary Information [file srep27215-s1.pdf]

Supplementary information

Title: Foci of cyclin A2 interact with actin and RhoA in mitosis

Authors: Abdelhalim Loukil[1], Fanny Izard[1], Mariya Georgieva[1,2], Shaereh Mashayekhan[2], Jean-Marie Blanchard[1], Andrea Parmeggiani[2,3] and Marion Peter[1,*]

Affiliations:

[1]Institut de Génétique Moléculaire de Montpellier
CNRS, Université de Montpellier
1919 route de Mende, 34293 Montpellier
France

[2]Dynamique des Interactions Membranaires Normales et Pathologiques
CNRS, Université de Montpellier
Pl. E. Bataillon, 34095 Montpellier Cedex 5
France

[3]Laboratoire Charles Coulomb
CNRS, Université de Montpellier
Pl. E. Bataillon, 34095 Montpellier Cedex 5
France

[*]**Contact:** Marion Peter
Phone: (+33)434359662
Fax: (+33)434359634
E-mail: marion.peter@igmm.cnrs.fr

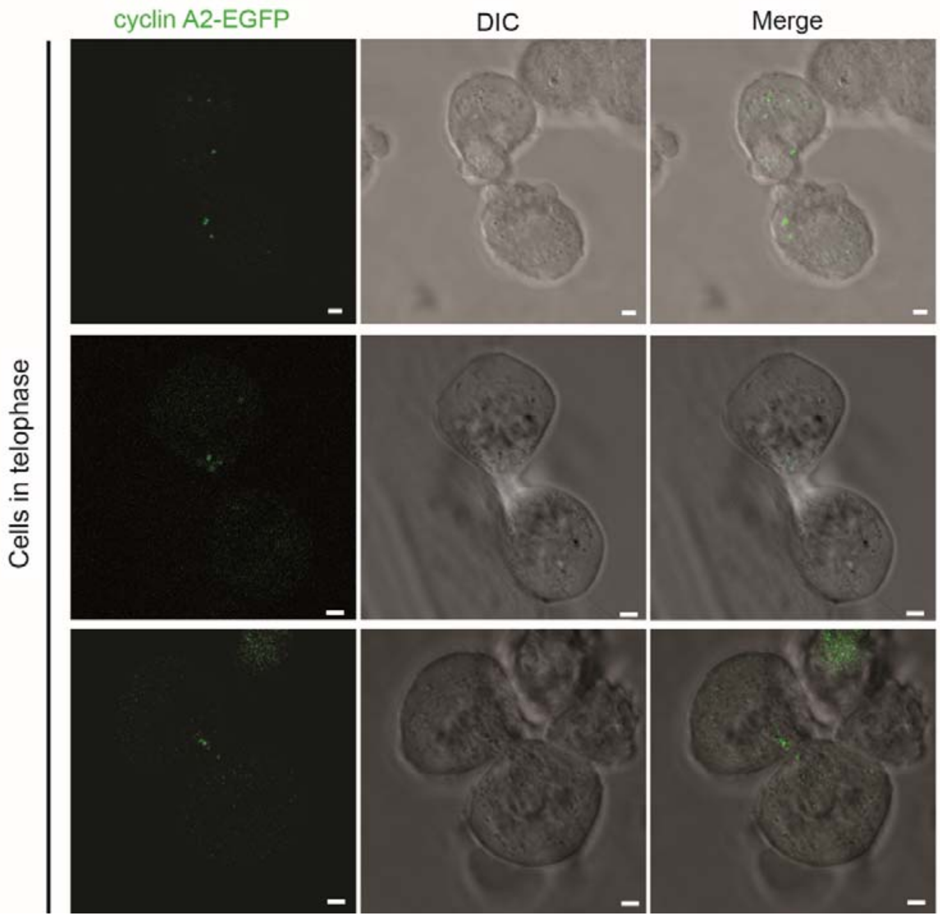

Supplementary figure S1. Cyclin A2-EGFP foci in telophase.

Live U2OS cells induced to express cyclin A2-EGFP, imaged in telophase. Left: EGFP images obtained by confocal microscopy. Center : DIC images. Right: merge images.

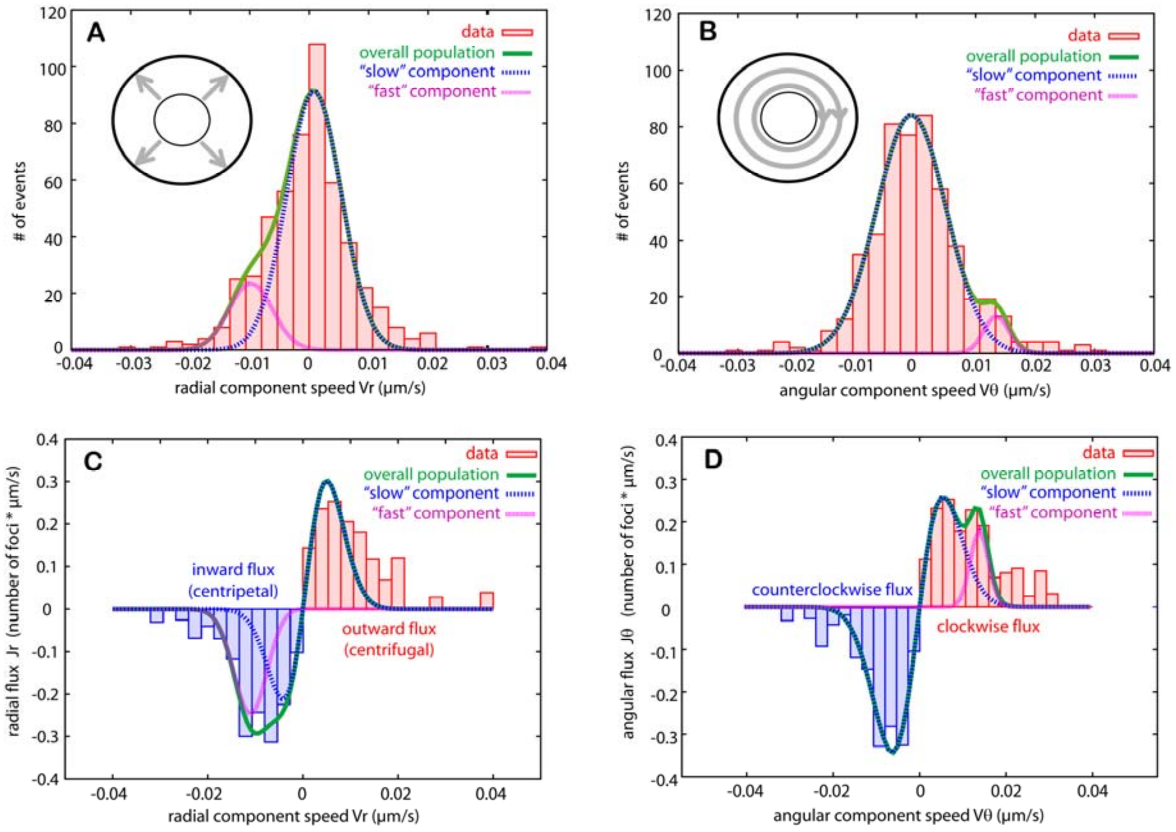

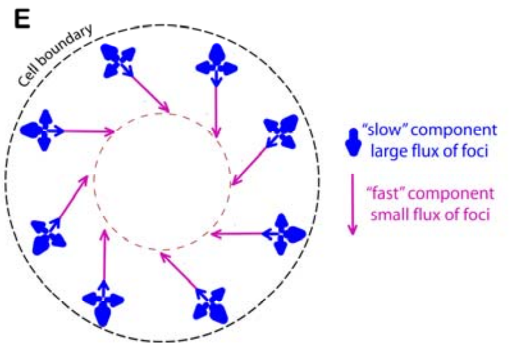

Table 2

"Fast" component	"Slow" component
Vr =(-9,8 ± 0,2)·10^{-3} μm/s	Vr =(0,8 ± 0,6)·10^{-3} μm/s
Vθ=(13,5±0,8)·10^{-3} μm/s	Vθ=(-0,8 ± 0,2)·10^{-3} μm/s

Supplementary figure S2. Foci speed and flux analysis.

A-B. Distributions of the radial (A) and angular (B) speeds V_r and V_θ and relative fits with Gaussian functions. Fits confirm the presence of a fast component of foci moving inward and clockwise (purple dotted line). Speeds are compatible with the MSD analysis (see Figure 2).

C-D. Distribution of the total fluxes of foci as a function of foci speeds. Fluxes are mostly distributed uniformly in space. Fast components (purple dotted line) seem predominantly oriented inward (C) and clockwise (D). Curves were directly obtained from the curve fit values in figures S2A and S2B.

E. Scheme of possible foci fluxes inside a mitotic cell. Some foci move fast along the inward and clockwise direction (purple arrows), while the others move more slowly in all directions (blue arrows).

Table 2. Typical values of foci speeds in inward/outward and clockwise/counter-clockwise directions as obtained by statistical analysis in figures S2A and S2B.

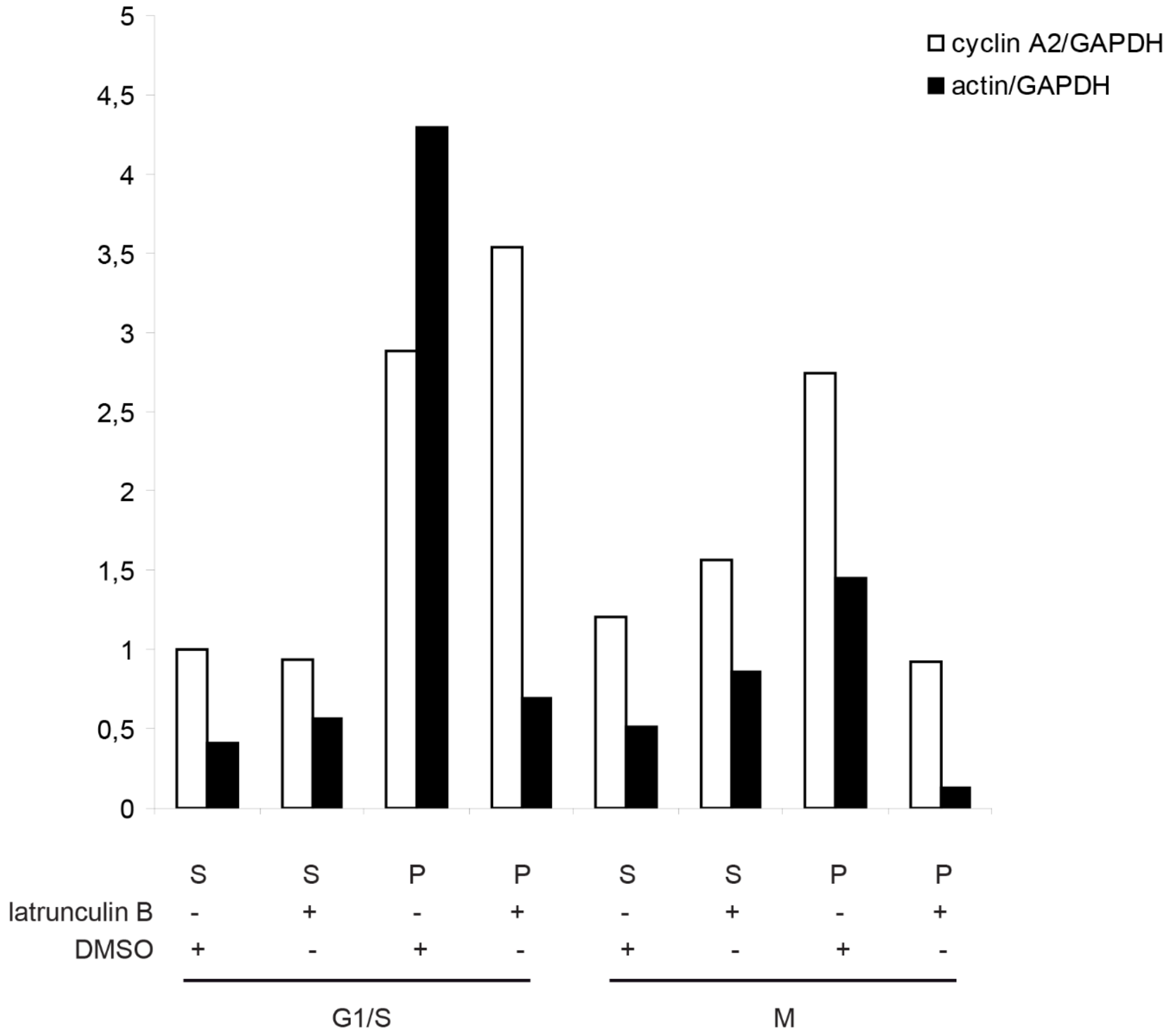

Supplementary figure S3. Actin depolymerisation experiment.

Quantification of western blots shown in figure 3A.

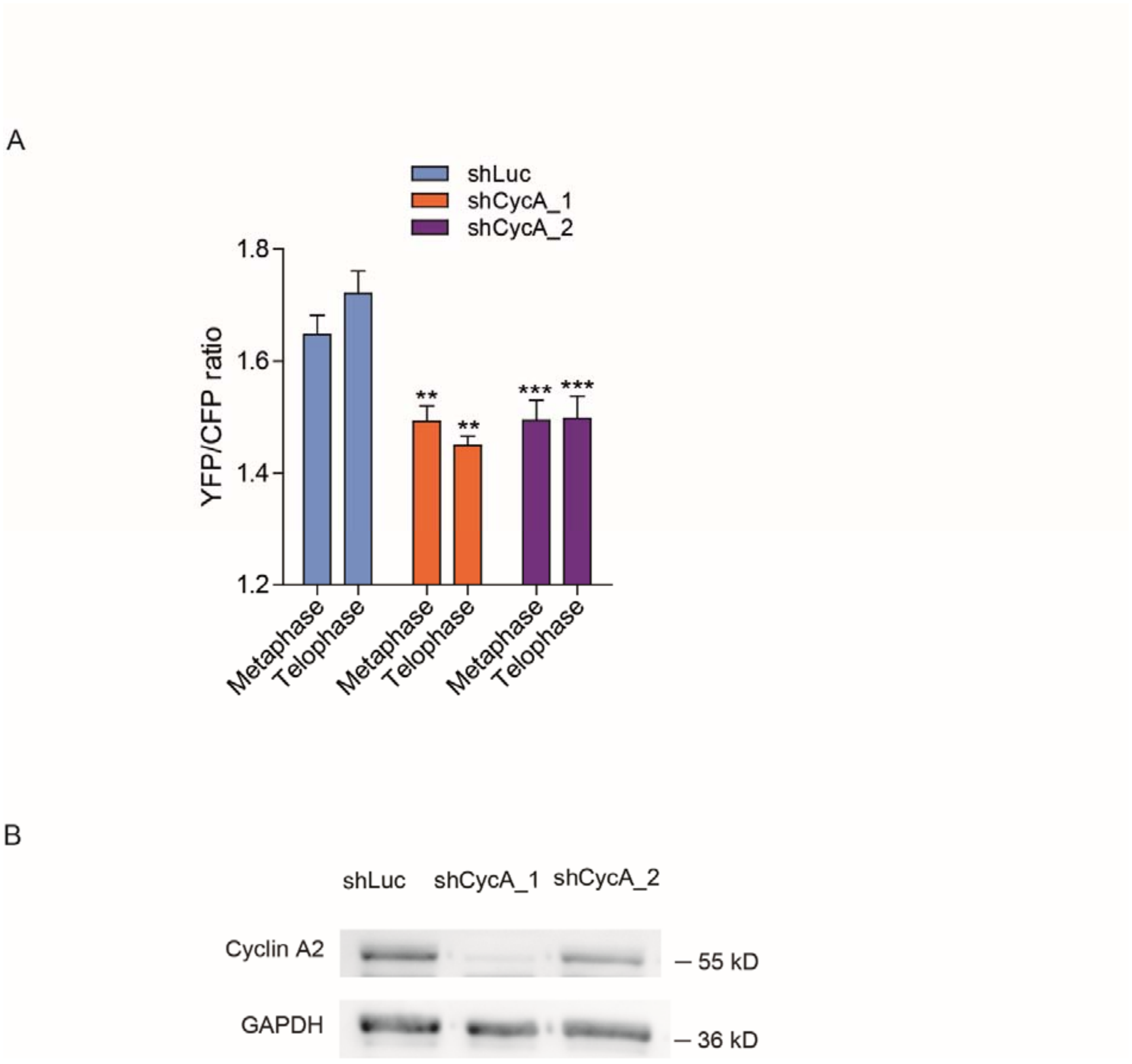

Supplementary figure S4. Effect of cyclin A2 shRNA.

A. NMuMG cells were infected with retrovirus carrying shRNA against luciferase (shLuc) or cyclin A2 (shCycA_1 or _2), transfected with pTRIEx-RhoA FLARE.sc Biosensor WT and imaged through mitosis. YFP/CFP intensity ratio values were collected from 30 random cell areas. Data represent the mean±s.e.m. Following comparison to shLuc expressing cells in the corresponding mitotic phase, P[shCycA_1, metaphase]=0.0019 ; P[shCycA_1, telophase]=0.0014 ; P[shCycA_2, metaphase]< 0.0001 ; P[shCycA_2, telophase]< 0.0001.

B. Western blot from total cell extracts of NMuMG cells following infection with retrovirus carrying shRNA against luciferase or cyclin A2, showing cyclin A2 levels. GAPDH is used as a loading control.

Supplementary movie 1. MCF-7 cell synchronised at G1/S transition by single thymidine block, microinjected with pEGFP-N1-cyclin A2 and observed 14h after release, in mitosis. Images obtained by two-photon excitation, with a FLIM detector. 20 s acquisitions with a pause of 20 s in between.

Foci trajectory analysis: mean squared displacement.

The analysis of foci trajectories allows quantifying their dynamics during mitotic progression. In figure 2C, we represent the estimate of the mean squared displacement in time (MSD)[1] measured from experiments. In figure 2C inset, we show the log-log plot of the MSD in time over all trajectories (in green). The line represents the fit using a power-law behaviour $MSD(t) \propto t^{\alpha}$. Fits interpolate very well the data up to 500 s (note that beyond 400 seconds statistics become relatively low and beyond 500 s there are only few trajectories available). Remarkably the exponent α obtained by the power law fit is close to the value 1.5, thus between purely diffusive (compare with the grey reference curve with power law ~ t) or purely ballistic motion (compare with the grey reference curve with power law $\sim t^2$). The value $\alpha \sim 1.5$ indicates that the foci motion can be super-diffusive. We can also argue that foci trajectories result from a coupling between Brownian and directed (active) motions. The MSD over all trajectories can be thus described by the typical behaviour in time of a Brownian motion coupled with directed motion, $MSD(t) = 4\ D_a t + v_a^2 t^2$, where D_a is the typical diffusion coefficient and v_a is the typical drift velocity of the foci.

The analysis has also allowed detecting two main classes of foci motile behaviours among all trajectories (figure 2C-D and table 1). About 29 out of 77 foci show a more dynamic movement characterised by higher diffusion coefficients and speeds than the remaining 48 foci. The fits reproduce well the behaviour of these "fast" and "slow" populations (represented respectively in red and blue). We then estimated their diffusion constants (D_f and D_s), and relative average speeds (v_f and v_s).

Diffusion constants for both populations are (very) small. We can estimate the intracellular effective viscosity from the classical Stokes-Einstein formula[2], $\eta = k_B T / 6\pi D r_f$, applied to a spherical particle where k_B is the Boltzmann constant, T the absolute temperature, D the diffusion constant estimated from data, and r_f the hydrodynamic radius of the foci. From images in figure 1A, we can evaluate r_f =250-400 nm from the full width at half maximum of the fluorescence profiles. Despite resolution limitations, these values allow estimating the order of magnitude of the medium effective viscosity for the slow population to $\eta_s \approx 1\text{-}1.5$ Pa·s,

while a lower bound of the medium effective viscosity for the fast population is $\eta_f \approx 0.15$-0.25 Pa·s. From these estimates, the effective viscosity of the medium is about 10^2-10^3 the water viscosity ($\eta_w = 10^{-3}$ Pa·s). Foci are therefore embedded in highly viscous medium compatible with very dense actin solutions[3] and/or membrane compartments[4] with large viscosity variations due to intracellular heterogeneities.

In particular, the fast population displays a typical speed of about 10 nm/s. This value is compatible with actin cytoskeletal reorganisation dynamics and intracellular motion[5]. All these results strongly suggest that foci motion can be related to the cytoskeleton activity and its reorganisation during the mitotic progression. Moreover, the presence of two populations of foci movements indicates potentially different interaction pathways of the foci with the cytoskeleton.

Foci trajectory analysis: global speed and flux analysis.

To investigate further these data, we also analysed the distributions of foci speeds and the related intracellular fluxes. In order to avoid any bias in our analysis, we considered again all data available, without using the results and information extracted from the previous data analysis.

First, we studied the radial and angular speed components, respectively V_r and V_θ (see below) for all foci (figures S2A and S2B). The statistical analysis suggests again the presence of two foci population components moving at different speeds. Estimates from fits (table 2) are compatible with the prior MSD analysis. Slow steps are the majority, but a relevant population of fast movements emerged from statistical analysis (figures S2A-S2B).

This information can be further exploited by providing an estimate of the average radial and angular fluxes of foci in cells for a given speed (figures S2C and S2D). The average flux of foci is indeed proportional to the foci number for each bin times the typical speed of the foci for the given bin. Figures S2C-S2D represent the fluxes and related fit curves as directly obtained from the speed analysis (without any additional adjustment) of figures S2A-S2B. The fast component is oriented inward (centripetal) and clockwise (purple arrows in figure S2E), while the slow components have a more pronounced tendency to move outward and counter clockwise (blue arrows in figure S2E). Nonetheless, these foci seem to move with different proportions in all directions.

Global flux analysis shows that foci tend to distribute in time homogeneously in the intracellular space. Overall, the foci fluxes are indeed practically equivalent in all directions parallel to the focal plane.

However, data indicate also that foci that move actively are preferentially translocating tangentially to the region where mitosis is taking place (purple arrows in figure S2E). This is an interesting and unexpected feature inferred by data analysis. This aspect could be relevant in future research to unveil the mechanisms for foci positioning during furrow cleavage formation and ingression.

Altogether these observations emphasise the potential interaction of the foci with the cytoplasm and in relation with the cytoskeleton dynamics, but further studies in this novel direction will be necessary.

Foci trajectory analysis: expressions of radial and angular velocities in Cartesian coordinates.

We provide here the expressions of the radial and angular components of the speed vector in Cartesian coordinates. For a given foci of Cartesian coordinates **r**=(r_x,r_y) and speed vector **V**=(V_x, V_y), one can get, by simple arguments of linear algebra and vectorial calculus, the radial speed component V_r and the angular speed component V_θ *via* the following expressions:

$$V_r = \frac{V_x r_x + V_y r_y}{\sqrt{r_x^2 + r_y^2}} \quad \text{and} \quad V_\theta = \frac{V_x r_y - V_y r_x}{\sqrt{r_x^2 + r_y^2}}$$

References

1. Gardiner, C. W. Handbook of Stochastic Methods for Physics, Chemistry and the Natural Sciences. *Springer series in synergetics* **13** (1985).
2. Howard, J. *Mechanics of motor proteins and the cytoskeleton.* Sinauer (2001).
3. Arrio-Dupont, M., Foucault, G., Vacher, M., Devaux, P. F. & Cribier, S. Translational diffusion of globular proteins in the cytoplasm of cultured muscle cells. *Biophys J* **78**, 901-907 (2000).
4. Haidekker, M. A. *et al.* New fluorescent probes for the measurement of cell membrane viscosity. *Chem Biol* **8**, 123-131 (2001).
5. Kohler, S., Schaller, V. & Bausch, A. R. Collective dynamics of active cytoskeletal networks. *PLoS One* **6**, e23798 (2011).
